# Herbivores Influence the Growth, Reproduction, and Morphology of a Widespread Arctic Willow

**DOI:** 10.1371/journal.pone.0101716

**Published:** 2014-07-21

**Authors:** Katie S. Christie, Roger W. Ruess, Mark S. Lindberg, Christa P. Mulder

**Affiliations:** The Institute of Arctic Biology, University of Alaska Fairbanks, Fairbanks, Alaska, United States of America; Lakehead University, Canada

## Abstract

Shrubs have expanded in Arctic ecosystems over the past century, resulting in significant changes to albedo, ecosystem function, and plant community composition. Willow and rock ptarmigan (*Lagopus lagopus, L. muta*) and moose (*Alces alces*) extensively browse Arctic shrubs, and may influence their architecture, growth, and reproduction. Furthermore, these herbivores may alter forage plants in such a way as to increase the quantity and accessibility of their own food source. We estimated the effect of winter browsing by ptarmigan and moose on an abundant, early-successional willow (*Salix alaxensis*) in northern Alaska by comparing browsed to unbrowsed branches. Ptarmigan browsed 82–89% of willows and removed 30–39% of buds, depending on study area and year. Moose browsed 17–44% of willows and browsed 39–55% of shoots. Browsing inhibited apical dominance and activated axillary and adventitious buds to produce new vegetative shoots. Ptarmigan- and moose-browsed willow branches produced twice the volume of shoot growth but significantly fewer catkins the following summer compared with unbrowsed willow branches. Shoots on browsed willows were larger and produced 40–60% more buds compared to unbrowsed shoots. This process of shoot production at basal parts of the branch is the mechanism by which willows develop a highly complex “broomed” architecture after several years of browsing. Broomed willows were shorter and more likely to be re-browsed by ptarmigan, but not moose. Ptarmigan likely benefit from the greater quantity and accessibility of buds on previously browsed willows and may increase the carrying capacity of their own habitat. Despite the observed tolerance of willows to browsing, their vertical growth and reproduction were strongly inhibited by moose and ptarmigan. Browsing by these herbivores therefore needs to be considered in future models of shrub expansion in the Arctic.

## Introduction

Climate warming in the Arctic has resulted in the rapid expansion of woody shrubs over the past half-century [1]–[4]. This shrub expansion has been likened to the melting of sea ice since shrubs (dark objects on the landscape) lower ground surface albedo, absorb heat, and accelerate snowmelt, thus creating a positive-feedback to climate warming [5], [6]. A process that will strongly interact with future climate warming to shape Arctic ecosystems is herbivory, which can significantly reduce the biomass of shrub species that would otherwise become dominant under warmer conditions [7]–[9]. For example, a study of vegetation changes along a sub-Arctic river found that herbivory greatly reduced the proportion of willows on the landscape while increasing the proportion of alder (*Alnus tenuifolia* Betulaceae) [10].

Deciduous shrubs growing in productive areas have had the most pronounced and rapid response to climate change, but also experience the greatest levels of herbivory [2], [11], [12]. Although the expansion of deciduous shrubs is known to be strongly regulated by herbivores [9], [13], [14] this plant functional group has also shown remarkable resilience to herbivory (15,16). The degree to which deciduous shrubs are regulated by herbivores will depend on the intensity of herbivory, site productivity, and the tolerance of the forage species.

Herbivores are capable of altering the morphology, productivity, and chemistry of preferred species [15], [16], which in turn influence the population dynamics of both plants and herbivores [17], [18]. In some plant-herbivore systems, herbivory results in an increase in quality, quantity, and/or accessibility of food, and these areas are called “grazing lawns” [15], [19]–[21]. Mechanisms causing this phenomenon include fertilization via urine and feces and changes to plant physiology and development that facilitate compensatory growth [22]. These high-quality foraging areas, maintained by repeated grazing, have a greater carrying capacity and support more animals than un-grazed areas [18]. This process is not limited to grazing systems; browsers of woody plants can also increase the palatability, accessibility, and biomass of their food resources [20], [23], [24]. However, not all plant-herbivore systems lend themselves well to the creation of grazing lawns. Depending on the intensity of herbivory, plant physiological and genetic constraints, and water and nutrient availability, plants can increase, decrease, or have the same aboveground productivity relative to un-grazed plants. These responses represent over-compensation, under-compensation, and exact compensation, respectively [22], [25].

In northern Alaska, moose (*Alces alces*) and ptarmigan (*Lagopus lagopus, L. muta*) concentrate in Arctic riparian areas where forage productivity is high and willows grow tall enough to exceed snow depth in winter (Figure S1). An important winter and spring food source for ptarmigan and moose in northern Alaska is feltleaf willow (*Salix alaxensis;* Salicaceae, Andersson), which is often the only willow species available for browsing in winter [26], [27]. Feltleaf willows establish on newly formed alluvial deposits, and their distribution is therefore tightly linked to fluvial dynamics of rivers. Over time and increasing distance from the riparian floodplain, feltleaf willows are replaced by later successional species such as Siberian alder (*Alnus viridis* subsp *fruticosa*), dwarf birch (*Betula nana*; Betulaceae), and other willows (e.g. *Salix lanata*, *Salix glauca*; [28]. High frequencies of browsing of willows in Arctic river valleys suggest that ptarmigan and moose may have a large cumulative impact on riparian shrub communities [29], [30].

Many species of willows are remarkably tolerant to mammalian browsing, and respond by producing shoots with increased biomass and nutritional quality after being browsed [16], [17], [31], [32]. These changes confer advantages to herbivores and can result in selectivity for previously browsed twigs [33], [34]. However, the effects of browsing by avian herbivores such as ptarmigan are not well understood, and may or may not be similar to those of mammalian browsing. Ptarmigan are highly abundant herbivores and congregate in large aggregations to feed on woody shrubs in the Arctic (flocks of tens of thousands have been observed [35]), and it is therefore important to define their impact on Arctic shrub communities. Ptarmigan feed predominantly on willow buds that are closest to the surface of the snow [30], [36], and typically remove the terminal buds, causing the shoot to die and new shoots to form from buds at the base of the branch [30]. This results in a highly complex, “broom-like”, architecture [30]. In addition to changes to architecture, browsing by ptarmigan may increase bud population growth rates, as observed by Tolvanen et al. [37] in muskox (*Ovibos moschatus*) -grazed willows. Increased bud production, in combination with broomed architecture may be beneficial for future ptarmigan browsing because the two processes result in higher concentrations of buds within easy reach of ptarmigan.

This study addresses three important questions. 1) What is the extent and intensity of browsing on feltleaf willows? 2) What are the mechanisms by which ptarmigan and moose influence the growth and reproduction of feltleaf willows? 3) Do ptarmigan and moose increase forage availability through browsing, thereby creating and maintaining “browsing hedges” in Arctic shrub ecosystems? We used stage-structured population models to quantify how the survival and production of new buds differ between browsed and unbrowsed willows, and how this in turn influences bud abundance, shoot and catkin production, and plant architecture.

## Methods

### Study area

We applied for and received permits to conduct our research on federal lands (NPS Permit# NOAT-2012-SCI-006; BLM Permit #FF095785). This study did not require the use of endangered or protected species. We selected two geographically distinct regions in northeastern and northwestern Alaska known to have ptarmigan and moose populations associated with willow thickets tall enough to exceed maximum snow depth. One study area was located along a 45 km segment of the upper Noatak River in the Noatak National Preserve (68.0°N, 158.0°W to 68.0°N, 159.2°W), which flows westward from the Brooks Range in the Gates of the Arctic National Park to the Chukchi Sea. The other study area consisted of a 157 km stretch of the Dalton Highway, between Galbraith Lake and Franklin Bluffs (68.5°N, 149.5°W to 69.7°N, 148.7°W). Four of the five Dalton Highway sites were adjacent to the Sagavanirktok River, a major river flowing north from the Brooks Range to the Beaufort Sea. The southern-most site consisted of a gravel bar adjacent to Galbraith Lake. Other than this site, both study areas were located on wide, braided sections of the rivers, which flow through glacier-carved valleys surrounded by rolling hills. The plant communities in both study areas were characterized by a band of tall shrubs, dominated by feltleaf willow on floodplains, lower terraces, or gravel bars adjacent to the river. Vegetation transitioned to shorter willows (e.g. *Salix lanata*, *Salix glauca*), dwarf birch, and Siberian alder further from the river's edge [28].

### Feltleaf willow transects

Twenty sites along the Noatak (10 sites) and Sagavanirktok Rivers (10 sites) were initially selected for sampling based on the presence of feltleaf willow stands. Of these, 5 sites from each study area were selected for the present study using systematic sampling with an initial random selection to determine whether the first or second of the 10 sites should be sampled, and alternating sites were sampled thereafter. Sites consisted of feltleaf willow stands varying in size from approximately 3 to 100 ha. At each of the 10 study sites, 30-40 feltleaf willow plants were randomly chosen, labeled, and marked with flagging tape in June 2011. We randomly chose feltleaf willows such that all ramets in the riparian zone had an equal probability of being sampled. When a willow was identified for sampling, a random branch was selected, marked, and assessed for browsing that occurred over the winter (2010–2011) and for type of browser (Figure S2). Occasionally, for very small willows, the entire ramet was measured. Herbivores could be identified by the browse marks left on the willow. Ptarmigan removed the buds and occasionally stripped the bark from willow shoots, whereas moose removed complete portions of shoots, leaving behind remnants measuring approximately 4–9 mm in diameter at the point of browse [29]. Hares typically leave a sharp 45° angle on browsed shoots. We quantified the number of buds removed by ptarmigan by counting distinct orange bud scars left on the shoot after bud removal. The number of buds remaining on each shoot, number of shoots browsed by mammals, and shoots that remained (unbrowsed shoots) were also counted. These measures of browse were used to estimate intensity of browsing (proportion of buds or shoots that had been removed). The entire branch, including remaining willow buds, catkins, shoots, and scars where buds had been removed was marked with color paint and mapped, so that the fate of buds and vegetative shoots could be determined the following spring (Figure S3).

Marked willow branches were re-visited and mapped in June 2012 to document browsing that occurred over the previous winter, and to record plant characteristics such as height, catkin production, shoot growth, and bud production (Figure S2). Surveys were conducted at the beginning of the growing season, and therefore measurements of shoot growth and bud production in 2012 represented the previous year's growth (2011 growing season). Annual shoot growth was quantified by measuring diameter and length of vegetative shoots produced the previous growing season (2011). It was possible to differentiate 2011 growth from current (2012) annual growth because the former was woody and brown and the latter light green. Marked willows were also measured for height, whether any shoot on the plant had been recently browsed by moose or ptarmigan (this was later used to calculate browsing frequency), and the percent of branches that were “broomed” (where >2 shoots originate from a single node on the branch). This is indicative of historic browsing intensity: a plant with many broomed branches had been subjected to several consecutive years of browsing by ptarmigan or moose [16], [30].

## Data analysis

### Response of willow growth to herbivory

We quantified plant response to browsing based on the frequency and intensity of browsing by ptarmigan and moose at each site. Browsing intensity was measured as the proportion of shoots browsed by moose, and the proportion of buds removed by ptarmigan. Occasional browsing by snowshoe hares (*Lepus americanus*) was identifiable by distinct browsing marks on willow shoots and these plants were discarded from the analysis.

We assessed the effects of ptarmigan and moose browsing on subsequent catkin production (2011) and shoot volume (2012; Figure S2). Mixed linear models were used to model fixed effects (browse status - “unbrowsed”, “ptarmigan-browsed”, or “moose-browsed”) and random effects (site) using package “nlme” in program R (R Development Core Team version 2.15.1). Plants that were browsed by both ptarmigan and moose were classified as moose-browsed because usually only a few buds had been browsed by ptarmigan below the point of browse by moose and the effect of ptarmigan was in large part negated by moose. For shoot volume, we wished to examine the plant response to browsing that took place over the winter of 2010–2011, so we measured the mean volume of all shoots per random branch and total volume of shoots produced during the 2011 growing season. Shoot volume was calculated using the equation for a cone, using length and diameter at the base of the annual growth from 2011. Willows that were browsed by moose the subsequent winter (2011–2012) were not included in the analysis because new vegetative growth, including buds, had been consumed and could not be measured. Catkins were counted in the spring of 2011 to quantify direct removal of catkin-producing buds by both browsers during the winter of 2010–2011.

We tested whether a willow's past exposure to browsing was related to a) decreased plant height and b) increased likelihood of re-browsing. We assumed that highly broomed plants were exposed to intensive browsing for multiple years in the past. Tests for the existence of a negative relationship between proportion of branches broomed and height were conducted using linear mixed effect models with proportion of branches broomed as the fixed effect and site as the random effect. The probability of browsing by a moose or ptarmigan given the willow's historic exposure to browsing was assessed using a mixed logistic regression, with a binary response variable (browsed/unbrowsed). The probability of browsing was modeled as a function of the proportion of branches that were broomed (fixed effect), and site (random effect). Data on brooming and browsing collected in spring 2012 were used for this analysis.

## Bud demographic modeling

Bud demography models are a useful tool for understanding the effects of browsing on woody plants at the individual and population level [37]. Stage-based matrix population models [38] were used to compare bud population dynamics of ptarmigan-browsed and unbrowsed willows. See Text S1 for a detailed description of this analysis. It was not possible to conduct a bud demography study for moose-browsed willows because buds could not be counted after browsing. We predicted that willows would compensate for bud loss from browsing by stimulating dormant buds to produce vegetative shoots (themselves bearing buds) and increasing rates of bud production. For browsed and unbrowsed plants, we estimated mean vital rates for each stage in the bud life cycle and used these to calculate bud population growth rates. These demographic rates influence the production of vegetative shoots, plant architecture, bud abundance, and future food availability for ptarmigan.

## Results

A total of 182 felt leaf willows in the Dalton and 190 willows in the Noatak study area were surveyed. Browsing by ptarmigan and moose were the most prevalent types of browsing observed in our study areas. Browsing by hares and rodents was occasionally observed, and winter browsing by muskox and caribou (*Rangifer tarandus*) was not observed. Ptarmigan browsing occurred more frequently than moose browsing. In 2011, 88.5±0.1% (SE) and 84.7±0.1% of feltleaf willows were browsed by ptarmigan in the Dalton and Noatak study areas, respectively. Browsing frequencies by ptarmigan were similarly high in 2012, at 87.4±0.1% in the Dalton and 81.6±0.1% in the Noatak study area. In the Dalton study area, browsing by moose increased from 16.5±0.1% of willows in 2011 to 36.8±0.1% in 2012. In the Noatak study area, moose browsing frequency was similar between years at 44.2±0.1% in 2011 and 42.6±0.1% in 2012. In the Dalton study area, 15.9±0.01% (2011) and 35.7±0.01% (2012) of willows were browsed by both herbivores. At the Noatak study area, 37.9±0.01% and 33.2±0.01% of plants were browsed by both herbivores in 2011 and 2012, respectively. Hare browsing was low and inconsistent between years. In the Dalton study area, hares browsed no willows in 2011 and 8.8±0.04% of willows in 2012, all of which occurred at a single site. In the Noatak study area, hares browsed 3.7±0.02% of willows in 2011 and no willows in 2012. The majority of willows survived for the duration of the study; only three out of 372 willows died. Of the branches examined for browsing, ten died between 2011 and 2012 surveys, three of which were browsed by hares. The remaining seven branches were unbrowsed and died of unknown causes. The distal portions of shoots that had been browsed by ptarmigan usually died.

Ptarmigan and moose browsing intensity remained fairly consistent across years and sites; ptarmigan typically removed over a third of buds on willow branches, and moose browsed almost half of all new shoots. Ptarmigan removed a mean of 37.1±2.4% (SE; Dalton) and 36.1±2.1% (Noatak) of buds in 2011 and 38.9±2.4% (Dalton) and 30.3±2.2% (Noatak) of buds in 2012. Bud removal by ptarmigan was focused on the terminal end of shoots grown the previous growing season, and a few buds often remained at the base of each browsed shoot. In 2011, moose browsed a mean of 45.0±4.5% of shoots per branch in the Dalton study area and 55.4±3.8% of shoots per branch at the Noatak. Browsing intensity was slightly lower in 2012; moose browsed 39.0±4.4% of shoots at the Dalton and 45.5±4.2% of shoots at the Noatak study area.

**Figure 1:**
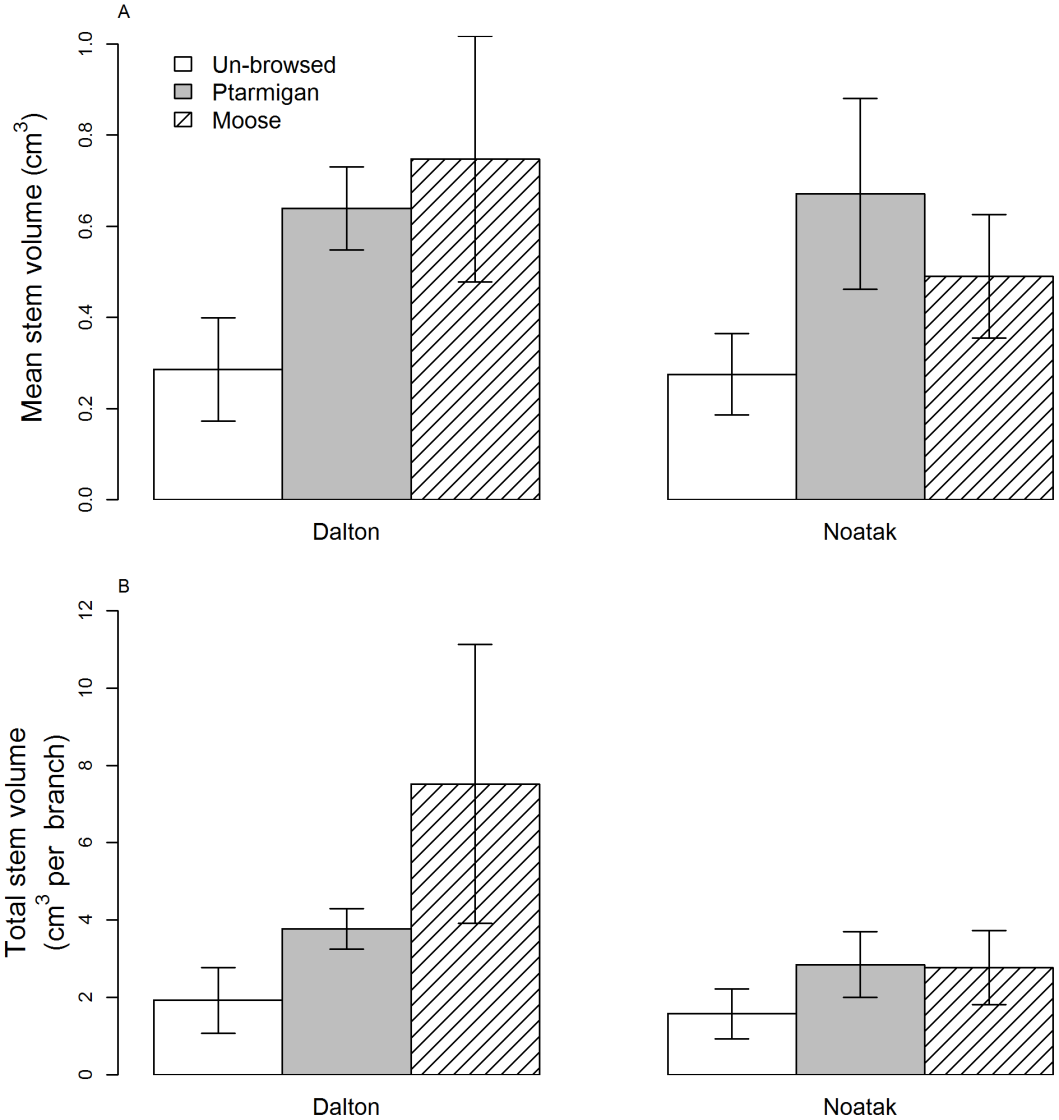
Mean (a) and total (b) shoot volume produced during the growing season by feltleaf willows (*Salix alaxensis*) that had been unbrowsed (U), browsed by ptarmigan (*Lagopus lagopus*, *L. muta*; P), or browsed by moose (*Alces alces*; M) the previous winter. Data were collected in 2012 from willows growing in the Noatak and Dalton study areas in northern Alaska. Error bars denote standard error.

Ptarmigan and moose-browsed willow branches produced shoots that were approximately twice (178–261%) as large in volume as unbrowsed willows (Figure 1a, moose: z-value  =  2.1, p = 0.03, n = 164, ptarmigan: z-value  =  2.6, p<0.01, n = 164). Browsed willow branches produced shoots that were longer and wider in diameter than shoots of unbrowsed branches. Total shoot volume (the sum of all individual shoots on a branch) was also significantly greater in browsed than unbrowsed branches for both herbivores (Figure 1b, moose: z-value  =  6.1, p<0.001, n = 164, ptarmigan: z-value  =  3.7, p<0.001).

**Figure 2:**
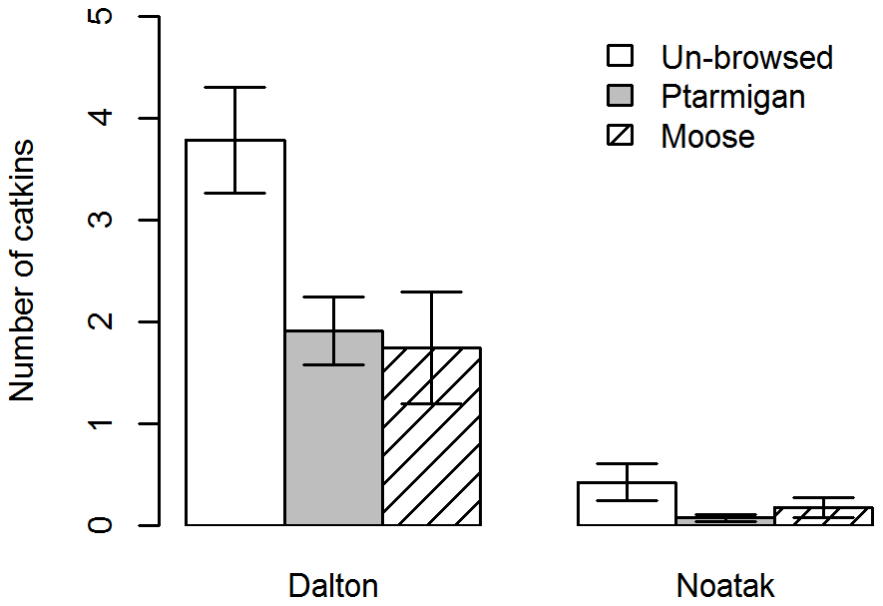
Number of catkins per branch of unbrowsed (U), ptarmigan-browsed (*Lagopus lagopus, L. muta*; P), and moose-browsed (*Alces alces*; M) feltleaf willows (*Salix alaxensis*) in the Dalton and Noatak study areas. Error bars denote standard error.

Both herbivores strongly influenced catkin production by directly removing catkin-producing buds prior to the spring reproductive period. Ptarmigan-browsed willows had 25–50% fewer catkins (depending on the study area) and moose-browsed willow branches had 54–59% fewer catkins than unbrowsed willow branches (moose: z-value  = −4.9, p<0.001, ptarmigan: z-value  = −5.5, p<0.001, n = 372, Figure 2). Willows in the Noatak study area produced substantially fewer catkins than those in the Dalton study area.

**Figure 3:**
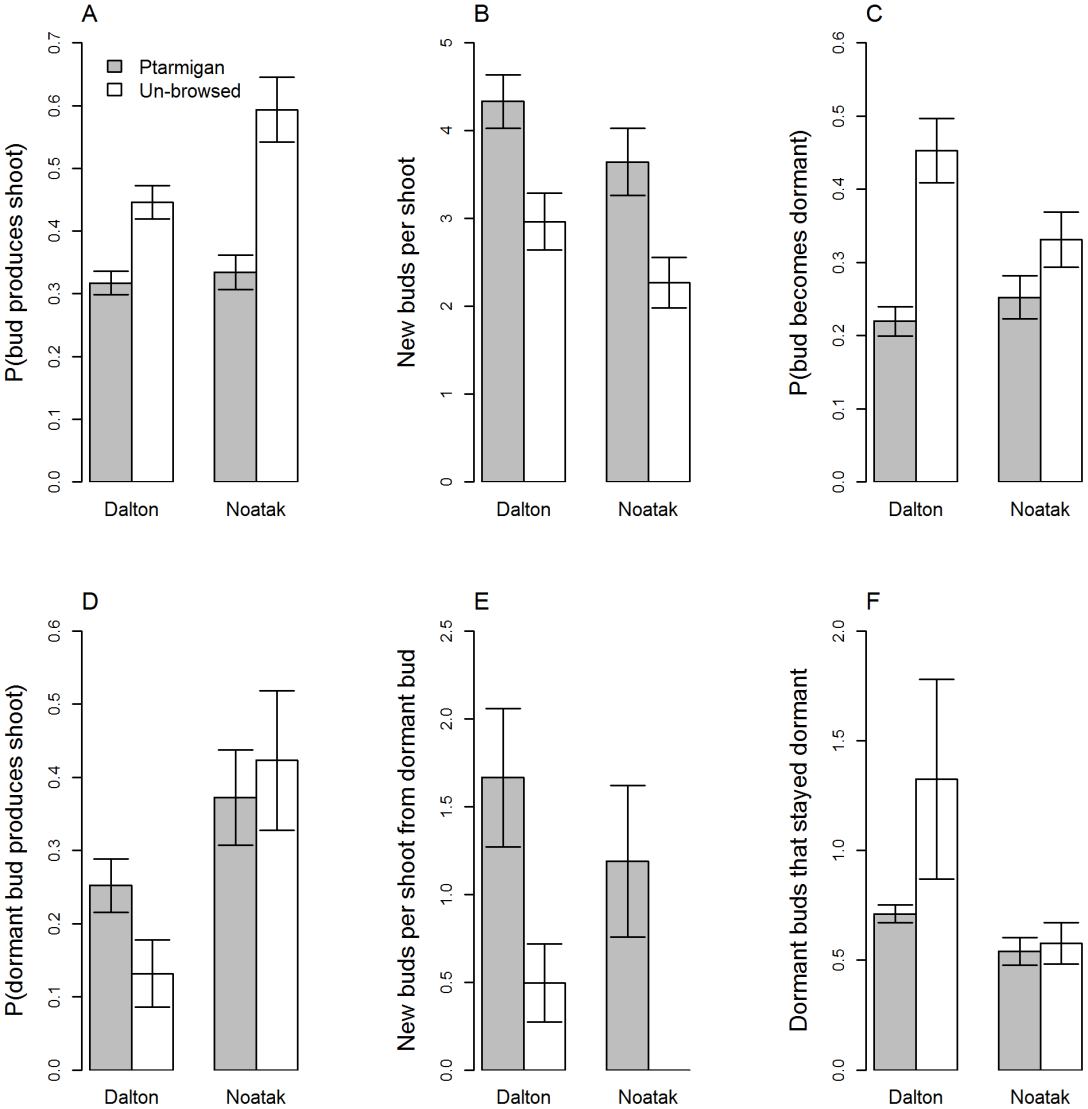
Vital rates (and standard errors) of ptarmigan (*Lagopus lagopus*, *L. muta*)-browsed and unbrowsed feltleaf willows (*Salix alaxensis*) in the Dalton and Noatak study areas. Figures A, C, and D reflect probabilities of buds transitioning from one state to another, whereas B, E, and F reflect numbers of buds per shoot. “Dormant buds” are adventitious buds on previous years' growth.

By activating axillary (at the base of the shoot) and dormant adventitious (embedded in the cambium of older parts of the plant) buds for vegetative shoot production and increasing the number of buds per shoot, ptarmigan-browsed willows maintained similar population growth rates (λ: the change in number of buds per branch over time) to their unbrowsed counterparts (Dalton: λ_unbrowsed_  = 1.49, λ_browsed_  = 1.37; Noatak: _λunbrowsed_  = 1.35, λ_browsed_  = 1.35). The LTRE (Life-table response experiment) analysis indicated that the largest contributor to variation in λ was the recruitment of new buds from dormant buds via the production of vegetative shoots (Dalton: 39% of total variation, Noatak: 51% of total variation; “F2”, Figure S4). Unbrowsed and ptarmigan-browsed willows had different bud fecundities and transition probabilities for both new and dormant buds (Figure 3). The probability of a bud producing a vegetative shoot was 41-77% higher for unbrowsed willows than ptarmigan-browsed willows in both study areas, and this is likely a direct result of bud removal by ptarmigan, i.e., browsed willows had fewer buds available for vegetative shoot production (β = −0.16, t-value  = −5.4, p<0.001, n = 125, Figure 3a). However, browsed willows produced 40–60% more new buds per vegetative shoot than unbrowsed willows (β = 1.29, t-value  = 3.3, p = 0.001, n = 125, Figure 3b). Fewer buds became dormant in browsed versus unbrowsed willows (β = −0.17, t-value  = −5.0, p<0.001, n = 125, Figure 3c). Dormant buds sprouted into vegetative shoots at higher rates in browsed versus unbrowsed willows at the Dalton study area but not at the Noatak, and the overall effect was not significant (β = 0.07, t-value  = 1.22, p = 0.23, n = 125, Figure 3d). At the Noatak, none of the vegetative sprouts that originated from dormant buds on unbrowsed willows survived to produce bud-bearing shoots (Figure 3e). New vegetative shoots produced from dormant buds bore more buds on browsed plants than unbrowsed plants (β = 0.97, t-value  = 2.10, p = 0.04, n = 125). Lastly, dormant buds stayed dormant at higher rates in unbrowsed versus browsed plants (β = 0.48, t-value  = −2.06, p = 0.04, n = 125, Figure 3f).

**Figure 4:**
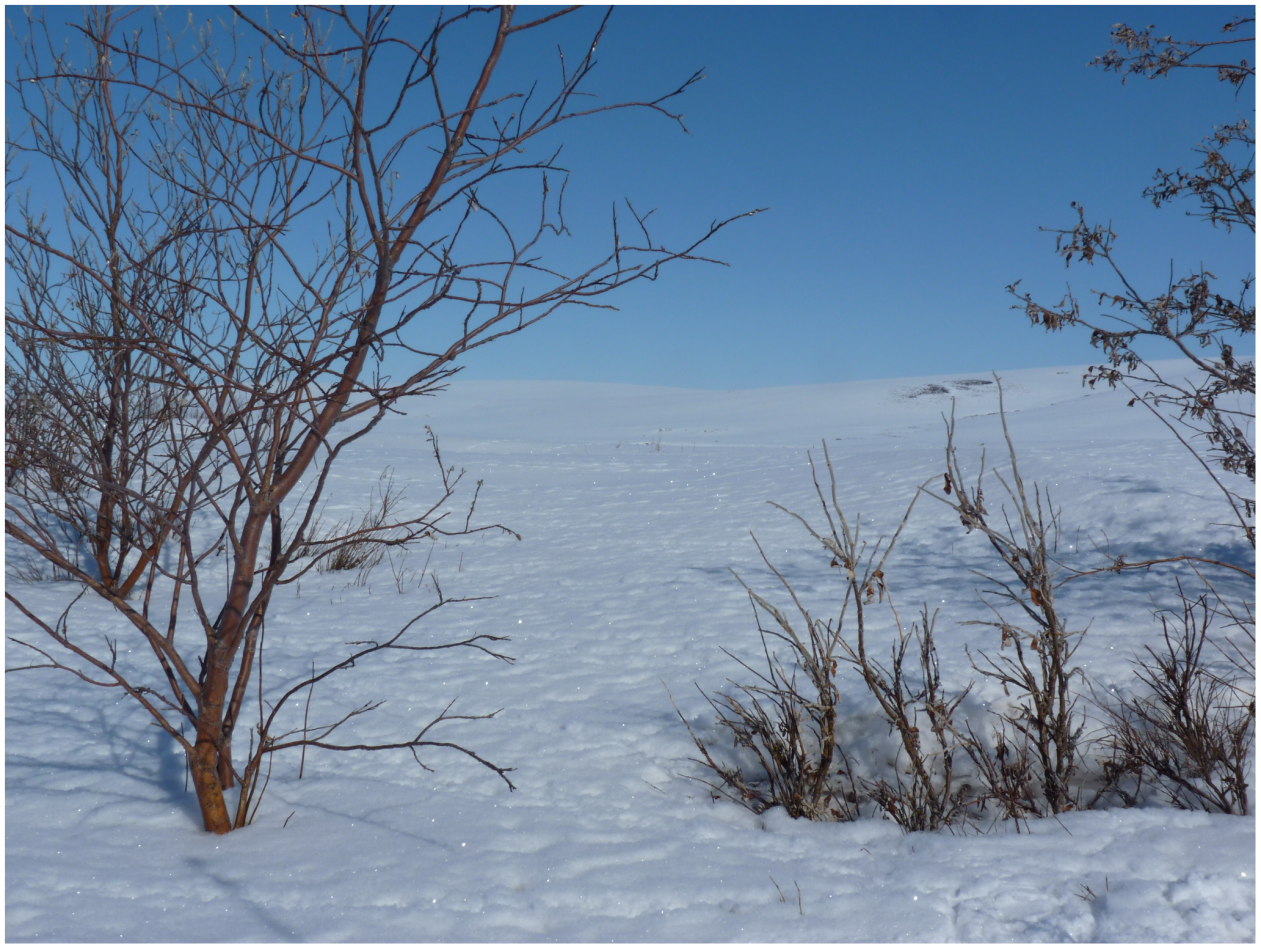
Un-broomed (left) and broomed (right) feltleaf willows (*Salix alaxensis*) in northern Alaska. Ptarmigan (*Lagopus lagopus, L. muta)* tracks are visible around the broomed willow.

**Figure 5:**
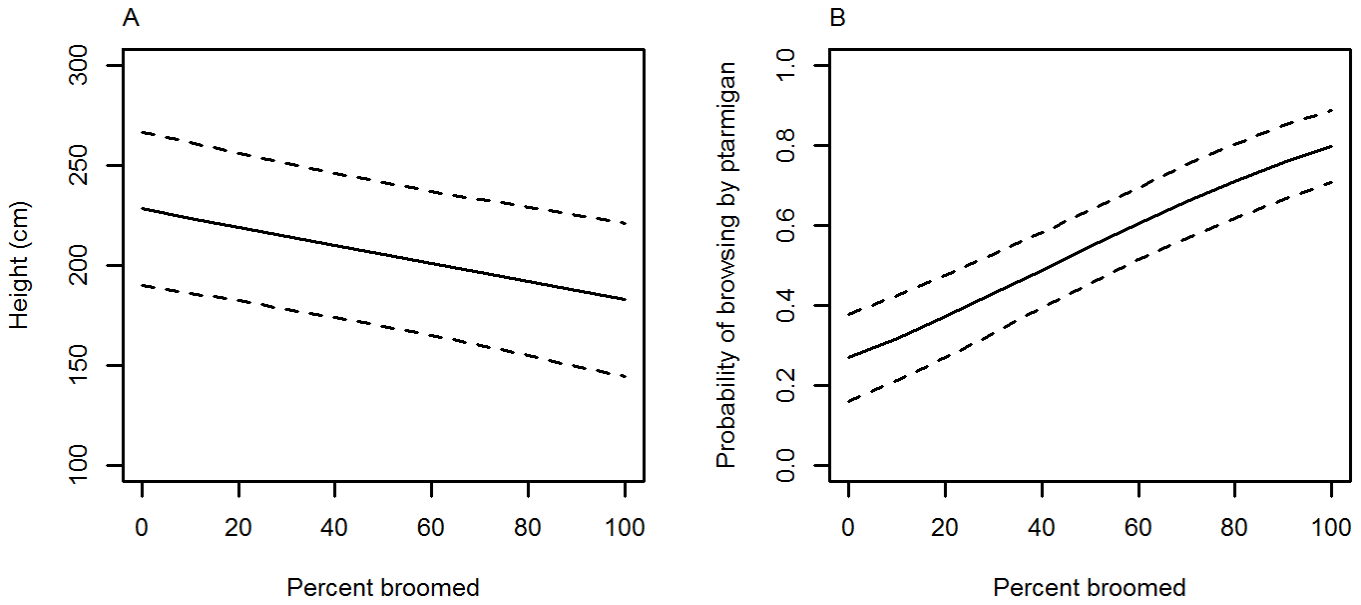
Relationship between percent of feltleaf willow (*Salix alaxensis*) branches that were broomed and plant height (a) and the probability of browsing by ptarmigan (b). Mixed models were used to assess the strength of relationships, with proportion of broomed branches as the fixed effect, and site as therandom effect. Dotted lines indicate upper and lower 95% confidence intervals.

Historic browsing, indicated by the proportion of broomed branches on the plant, was negatively related to total plant height (β = −0.46±0.13, t-value  = −3.39, p<0.001, n = 356) such that a heavily-broomed willow's height was reduced by 20% compared to an un-broomed willow (Figure 4, 5a). The probability of being browsed by ptarmigan increased significantly with the proportion of branches that were broomed (Figure 5b; z-value  = 5.5, p<0.001, n = 348), whereas no such relationship existed for moose (z-value  = 0.88, p = 0.38, n = 348).

## Discussion

Ptarmigan and moose strongly influenced willow biology in two geographically disparate regions of northern Alaska. Feltleaf willows responded to browsing by activating axillary and adventitious buds, and by producing long shoots with many buds. Repeated browsing resulted in short, structurally complex plants. Ptarmigan (but not moose) appeared to feed more frequently on these willows, which produced a food resource (buds) that was both accessible and concentrated. Early successional feltleaf willow stands provide optimal habitat for ptarmigan and moose not only because they grow tall enough to exceed snow depth in winter, but also because they are highly tolerant of herbivory [17], [32].

Willows that were browsed by ptarmigan or moose produced more than twice the volume of annual shoots compared with unbrowsed willows, indicating that feltleaf willows over-compensated for herbivory. Moderate herbivory can result in exact or over-compensation when plants have adequate access to nutrients and water and are inherently fast growing [19], [39], [40]. Feltleaf willows adhere to these characteristics and have been known to compensate for moderate levels of herbivory by snowshoe hares [17], [32]. The production of large shoots after browsing is adaptive because it allows the plant to a) quickly grow to escape herbivory; b) produce vegetative shoots and leaves for photosynthesis; and c) increase bud production to replace buds lost to browsing. Willows appear to respond similarly to ptarmigan and moose browsing, because both browsers remove terminal and distal axillary buds and cause shoot die-off and shoot loss, respectively.

By removing distal axillary buds that would otherwise become catkins, ptarmigan and moose interfere with sexual reproduction. This in turn is likely to hinder the ability of feltleaf willows to colonize areas such as newly formed alluvial surfaces, and may make them less competitive with other plants. Pollen and seed viability of Siberian alder, dwarf birch, and willows (*Salix* spp.) is expected to increase in the future as temperatures rise [41]. However, for highly palatable willows, the consumption of catkins by herbivores will likely reduce their ability to compete with other, less-preferred species.

Ptarmigan-browsed and unbrowsed willows had similar bud population growth rates, but these were maintained through different pathways. An unbrowsed willow branch will gradually elongate as terminal and distal axillary buds develop into shoot primordia during the growing season [42]. Leaves are distributed evenly along shoots and new buds develop adjacent to leaf petioles at the end of the growing season. By removing terminal and distal axillary buds, ptarmigan stimulate willows to activate proximal axillary and dormant adventitious buds to produce tissue required for photosynthesis and future bud production. This activation of axillary and adventitious buds, combined with the increased numbers of buds produced per vegetative shoot, allows willows to maintain bud populations at similar levels to unbrowsed plants. Although we were unable to directly measure the effect of moose browsing on bud demographic rates, we suspect that a similar process occurs due to the removal of shoots bearing terminal and distal axillary buds, as observed in other studies of mammalian browsing [37], [42].

The repeated removal of terminal buds by ptarmigan creates a broomed structure and constrains branch elongation, ultimately reducing the height of the willow, similar to how reindeer reduce the height of willows in Finnish Lapland and Siberia [8], [43], [44]. A key consequence of the altered architecture of browsed willows, combined with greater numbers of buds per shoot, is an increase in food availability for ptarmigan. Short, broomed willows are more accessible to ptarmigan, which prefer to feed on buds close to the snow [30], [36]. After several years of ptarmigan browsing, willows become “hedged” just above average snow level, providing optimal food accessibility for ptarmigan in future average snow years [30]. By increasing the quantity and accessibility of available forage in future years, flocks of ptarmigan may be creating “browsing hedges” analogous to grazing lawns maintained by ungulates [15], [25], sea turtles [45] and geese [18], [21]. In years of higher than average snow fall, willows may become buried and protected from browsing, whereas in years of lower than average snowfall, more willow branches become available for browsing. Food availability for ptarmigan is therefore strongly related to snow conditions of a given year. Whether these willows confer a nutritional advantage to ptarmigan in the form of increased quality of buds [15], [20] is beyond the scope of this paper and worthy of further investigation.

The greater volume of tissue produced by moose-browsed willow branches suggests that moose are also capable of increasing the quantity of their own food source. The compensatory response that we observed in feltleaf willows is consistent with observations of this species' response to mammalian browsing in boreal ecosystems [33], [46]. Moose, however, did not show a preference for previously browsed (broomed) willows. In our study, broomed willows had been exposed to a combination of ptarmigan and moose browsing in the past. The architectural complexity of highly broomed willows, which consisted of clusters of both live and dead (ptarmigan-browsed) shoots, may have restricted access to live shoots by moose [29]. Moose show a preference for woody plants with fewer, larger shoots, allowing for higher harvest rates [47], [48], and discriminate against well-defended shoots [34], [49]. Thus, by altering the architecture of willows, ptarmigan may reduce forage accessibility to moose. It is also possible that moose are deterred by secondary metabolites produced in the shoots of heavily broomed willows; feltleaf willows are known to produce less palatable twigs in response to severe browsing by snowshoe hares [50].

Due to the observational nature of this study, it is necessary to consider alternative explanations for the observed differences between browsed and unbrowsed willows. For example, ptarmigan and moose may have chosen to browse willows with greater access to resources and/or inherently faster growth rates than unbrowsed willows. Furthermore, the architecture of willows could potentially be influenced by winter abrasion and desiccation [50]. However, if this were the case with feltleaf willows, we would expect to see dead or broken branches with no signs of browsing, which we did not. We expect that severe winter conditions may be more important in limiting the growth of shrubs that occur on exposed ridgetops than those growing in protected river valleys [44]. Nevertheless, a simulated browsing experiment would help to tease apart the effects of browsing from other potentially confounding factors on willow growth and architecture.

In this study, we examined the growth and bud production at the branch-level rather than the entire plant. This was necessary for efficiency of data collection and also because few willows in our survey area were completely unbrowsed. Some of the unbrowsed willow branches were therefore attached to willows that had experienced low-level browsing. The fact that we observed such strong differences between the two branch types reflects the tendency of branches to operate as separate modular units within the plant, with correspondingly distinct physical and chemical characteristics [34].

A large proportion of feltleaf willows in our study areas were browsed by ptarmigan, and browsing by this herbivore was at least three times more prevalent than browsing by moose. Ptarmigan browsed much higher proportions of buds in our study areas (30–40%) than in Finland, where only 6% of willow buds were browsed despite the fact that ptarmigan were at the peak of their cycle [36]. The high frequency and intensity of browsing observed at our study sites reflect the importance of considering the effects of browsing by this small avian herbivore on Arctic shrub ecosystems in Alaska and perhaps elsewhere in North America. Surveys of spring ptarmigan distribution in northern Alaska indicated that shrub patches associated with small and large river drainages and areas with snow-free ground had a high probability (>85%) of being occupied by ptarmigan [51]. The degree to which ptarmigan populations in the study area fluctuate is unknown, although surveys by Irving et al [35] over the course of 15 years suggest that they do not cycle in northern Alaska, as they do in other parts of their range. Moose generally occur in low densities in northern Alaska, and concentrate in large river drainages with tall shrubs that exceed snow depth [52]. Although fewer willows were browsed by moose than by ptarmigan in our study areas, moose removed large amounts of tissue (45–55% of shoots per branch) and therefore also need to be considered as important Arctic herbivores.

Feltleaf willows were highly tolerant of herbivory and produced twice the volume of current annual growth relative to unbrowsed willows. This species of willow is in an optimal position to compensate for herbivory due to its inherently fast growth rate and tendency to grow on river floodplains, where frequent flooding provides access to water and nutrients. Despite its ability to tolerate browsing, the feltleaf willow experienced severely reduced reproductive output, and over the long term, distinctly altered height and architecture. By “pruning” willows on an annual basis, ptarmigan and moose prevent them from reaching their full reproductive and physical potential. This in turn increases the susceptibility of willows to further attack, thereby benefitting ptarmigan populations. Although deciduous shrubs are known to be highly resilient to herbivory [53], [54], repeated pruning by herbivores is likely to curtail their expansion in the Arctic and may facilitate the spread of less palatable species.

## Supporting Information

Figure S1 **Willow ptarmigan (** ***Lagopus lagopus*** **) near a felt-leaf willow (** ***Salix alaxensis*** **) stand in northeastern Alaska.**
(JPG)

Figure S2 **Timing of browsing and feltleaf willow (** ***Salix alaxensis*** **) growth in relation to timing of measurements.**
(TIF)

Figure S3 Map of felt-leaf willow branch first visited in June 2011 (left) and subsequently re-mapped in June 2012 (right). +-9**
(TIF)

Figure S4 **Retrospective contributions of matrix elements to variance in bud population growth rates of feltleaf willow (** ***Salix alaxensis*** **).** F1 is the production of new buds from first-year buds, F2 is the production of new buds from dormant buds, T1 is the probability of transition from first-year bud to a dormant bud, and T2 is the probability that a dormant bud will stay dormant. Positive values reflect an increase in the matrix element in ptarmigan (*Lagopus lagopus, L. muta)* -browsed compared to unbrowsed willows.
(TIFF)

Text S1 **Methods used to construct bud matrix population models for browsed and unbrowsed willows and figure depicting the life cycle of buds and vegetative shoots.**
(DOCX)
