# Supplementary figures and images for: Herbivores Influence the Growth, Reproduction, and Morphology of a Widespread Arctic Willow
Source: PLoS One. 2014 Jul 21;9(7):e101716. doi: 10.1371/journal.pone.0101716 (PMC4105470; doi:10.1371/journal.pone.0101716)

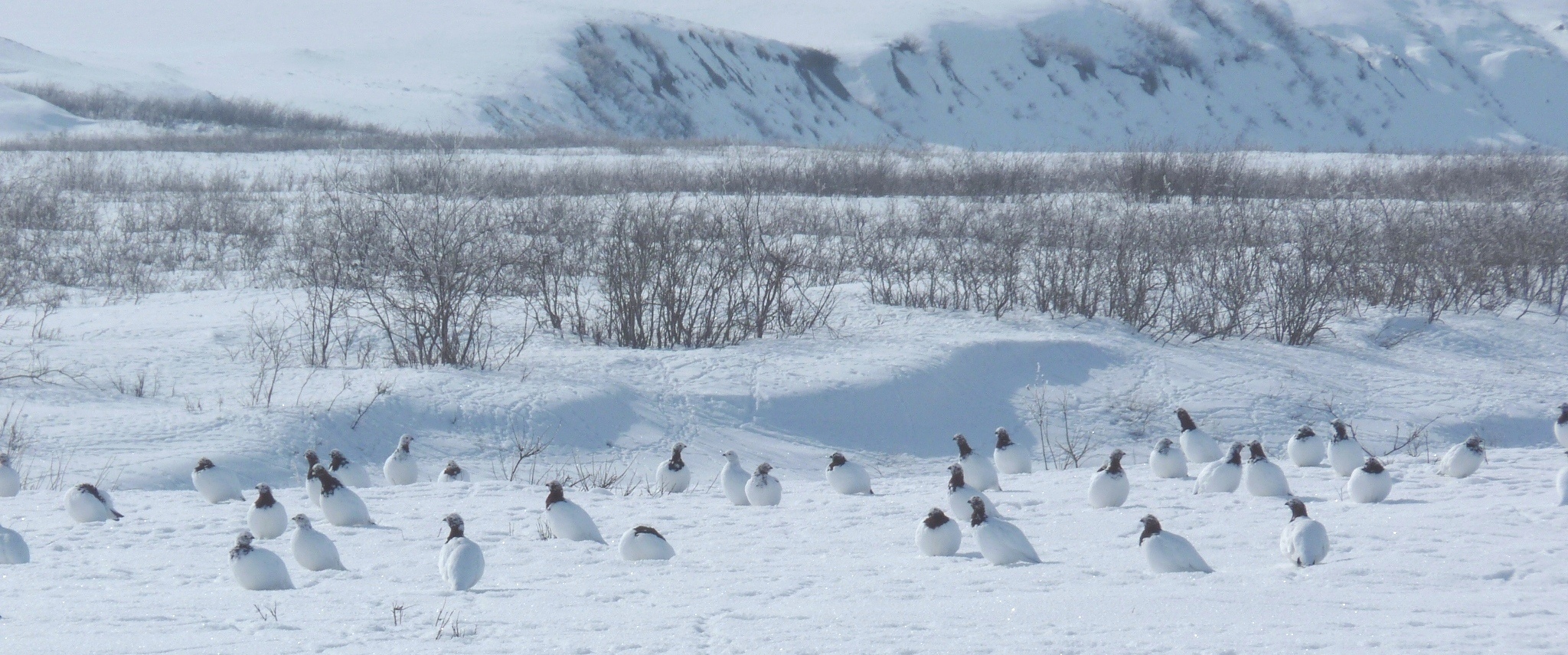

Supplement: Figure S1 — Willow ptarmigan ( Lagopus lagopus ) near a felt-leaf willow ( Salix alaxensis ) stand in northeastern Alaska. (JPG) [file pone.0101716.s001.jpg]

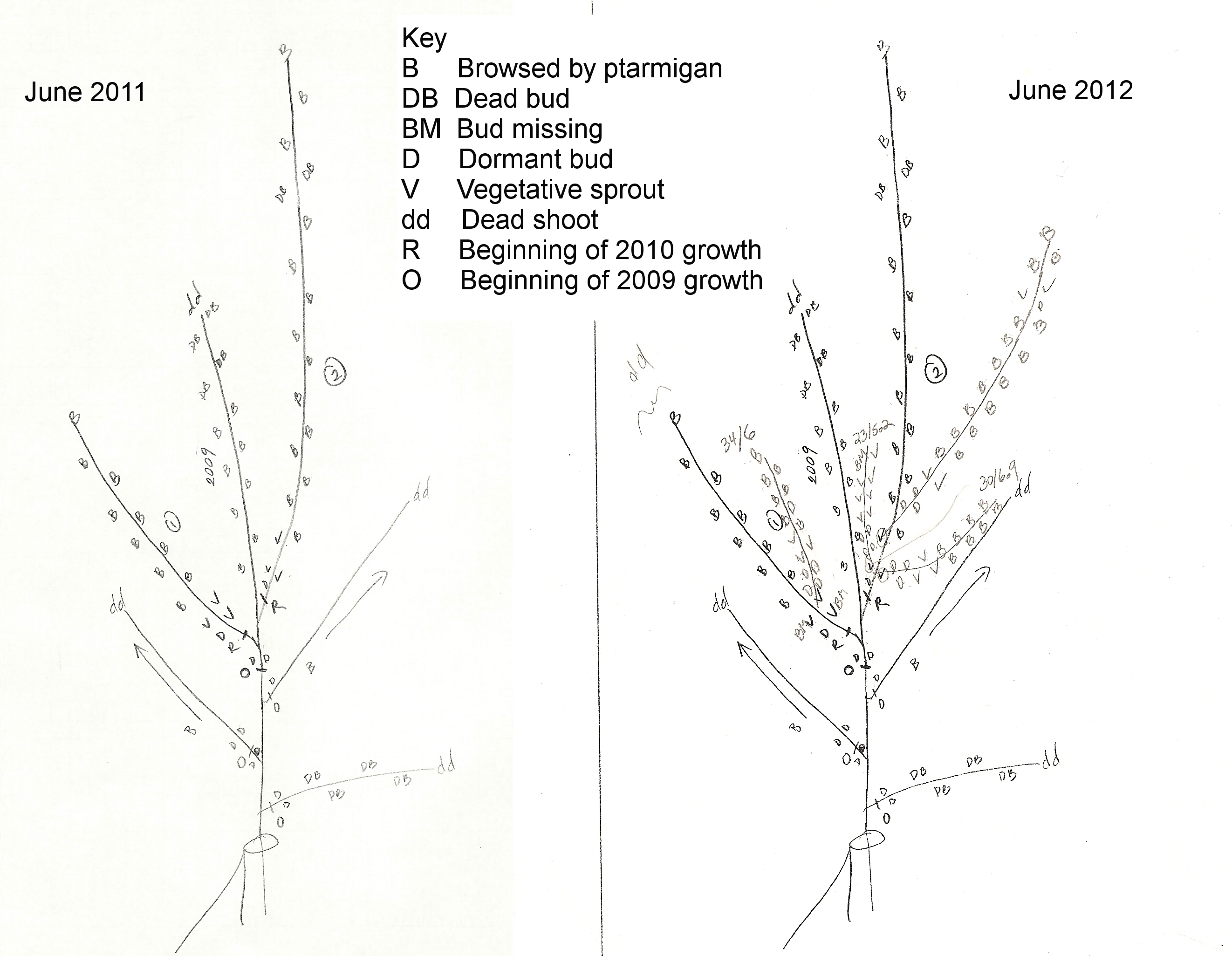

Supplement: Figure S3 — Map of felt-leaf willow branch first visited in June 2011 (left) and subsequently re-mapped in June 2012 (right). +-9** (TIF) [file pone.0101716.s003.tif]

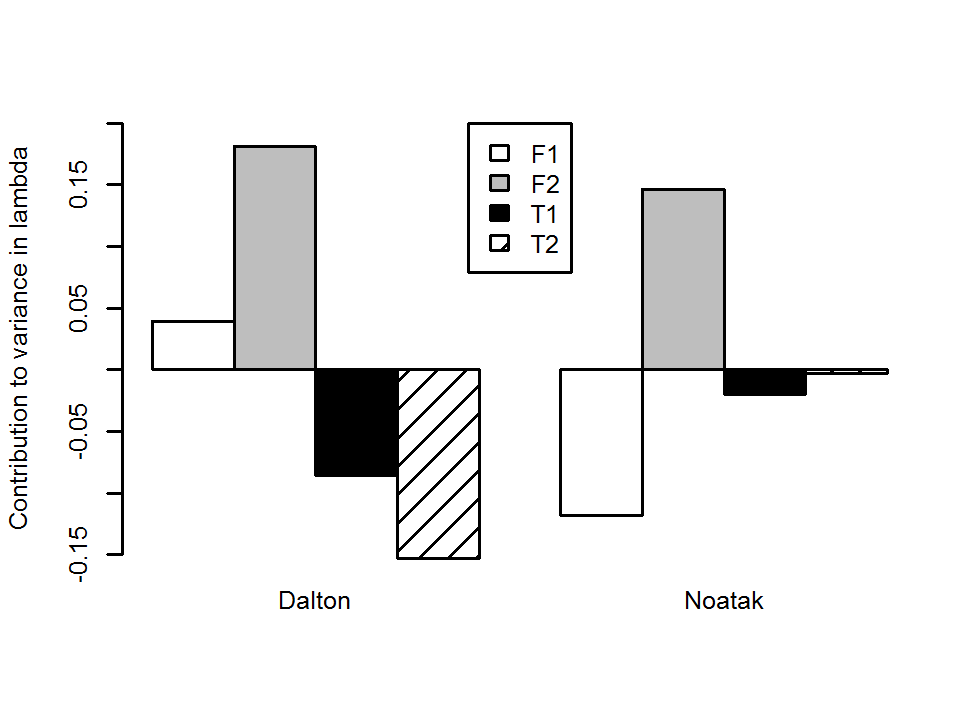

Supplement: Figure S4 — Retrospective contributions of matrix elements to variance in bud population growth rates of feltleaf willow ( Salix alaxensis ). F1 is the production of new buds from first-year buds, F2 is the production of new buds from dormant buds, T1 is the probability of transition from first-year bud to a dormant bud, and T2 is the probability that a dormant bud will stay dormant. Positive values reflect an increase in the matrix element in ptarmigan (Lagopus lagopus, L. muta) -browsed compared to unbrowsed willows. (TIFF) [file pone.0101716.s004.tif]
