# Supplementary material for: Herbivores Influence the Growth, Reproduction, and Morphology of a Widespread Arctic Willow
Source: PLoS One. 2014 Jul 21;9(7):e101716. doi: 10.1371/journal.pone.0101716 (PMC4105470; doi:10.1371/journal.pone.0101716)
Supplement: Text S1 — Methods used to construct bud matrix population models for browsed and unbrowsed willows and figure depicting the life cycle of buds and vegetative shoots. (DOCX) [file pone.0101716.s005.docx]

Text S1. Methods used to construct bud matrix population models for browsed and un-browsed willows and figure depicting the life cycle of buds and vegetative shoots.

Fecundities and transition probabilities (F1, F2, T1, T2; defined below) were calculated for two different stages: recently produced buds (hereafter “new buds”) and buds that were produced the previous growing season (“dormant buds”; Figure 1). New buds differed from dormant buds by their larger size, fuzzy outer covering, and more apical position on the stem (dormant buds tended to be very small and were positioned at the base of the stem). The production of new buds (F1) from buds was the product of the number of vegetative shoots produced per original bud and the number of new buds produced per vegetative shoot. The production of new buds from dormant buds (F2) was the product of the number of vegetative shoots produced per dormant bud and the number of new buds produced per vegetative shoot. The probability that a bud would transition into a dormant bud (and not become browsed, a catkin, or a vegetative shoot) was determined by dividing the number of buds that became dormant by the total number of buds produced the previous year; T1). The probability that a dormant bud would stay dormant (T2) was calculated as the number of dormant buds that stayed dormant divided by the total number of dormant buds present on the stem the previous year (Fig. C1). Mean vital rates (fecundities and transition probabilities) were compared between browsed and un-browsed willows using linear mixed models in program R with browsed/unbrowsed modeled as a fixed effect and site as a random effect. Using these vital rates, we constructed matrices for each browsed and un-browsed willow branch. An average matrix was calculated using the mean parameters for each type of willow branch in each study area, and lambda was calculated as the maximum eigenvalue for each average matrix. We subsequently calculated retrospective contributions of each parameter (F1, F2, T1, T2) to differences in lambda between browsed and un-browsed plants with a life table response experiment (Caswell 2001) using package “popbio” in program R.

References

Caswell, H. 2001. Matrix Population Models, 2nd edition. Sinauer Associates Inc., Sunderland.


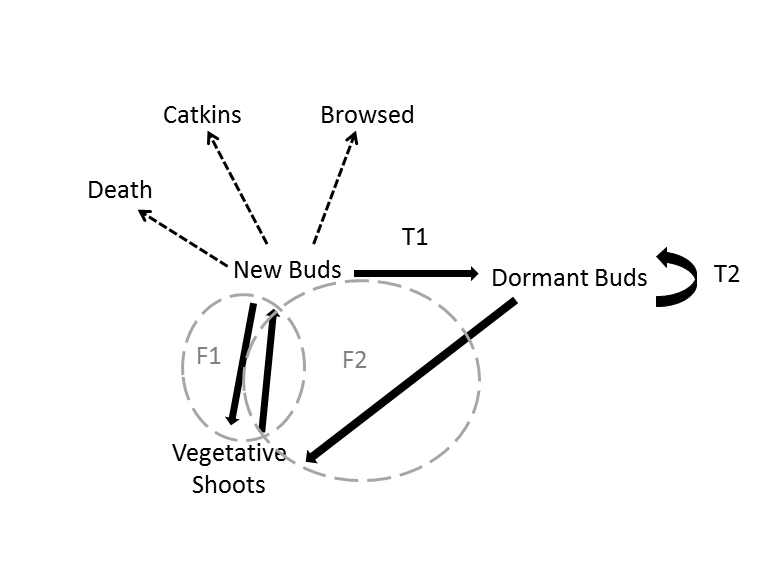


FIG. 1. Life cycle of buds and vegetative shoots of the felt-leaf willow (*S. alaxensis*) showing fecundities (F1, F2) and transition probabilities (T1, T2). Grey dashed lines indicate that fecundities are are the product of two lower-level parameters (number of vegetative shoots produced per bud and number of buds subsequently produced per vegetative shoot).
